# From their eyes: Deaflympic athletes' and coaches' perspectives on mental training

**DOI:** 10.3389/fspor.2025.1613833

**Published:** 2025-06-17

**Authors:** Alon Markov-Glazer, Anne-Marie Elbe, Rainer Schliermann

**Affiliations:** ^1^ Faculty of Social and Health Care Sciences, Regensburg University of Applied Sciences, Regensburg, Germany; ^2^ Faculty of Sport Science, Chair of Sport Psychology, Leipzig University, Leipzig, Germany

**Keywords:** Deaf sport, Deaf culture, sign language, sport psychology, psychological skill training (PST)

## Abstract

**Introduction:**

Sport psychology research has largely overlooked athletes with hearing impairments competing in Deaflympic sports, the designated elite sporting movement for this population. This study therefore explored Deaflympic athletes' and coaches' perspectives on mental training in the elite Deaf sports.

**Methods:**

A qualitative approach was used, involving six focus group interviews with 23 athletes (*M*_age_ = 33.09; 26.1% female) and four coaches from individual and team Deaf sports. Data were analyzed using reflexive thematic analysis.

**Results:**

Three themes emerged: sport psychology consultation, the influence of visual orientation on psychological skills and demands, and Deaf sport culture and communication. While participants expressed strong interest in sport psychology, engagement with professionals was limited by accessibility issues, lack of sign language-fluent consultants, and structural barriers. Deaf athletes reported adapting some techniques to match their visual-spatial orientation and both advantages and challenges of visual reliance in sports. Distinct communication dynamics between native signers and spoken-language users within Deaf teams were also revealed.

**Discussion:**

These findings highlight the importance of culturally sensitive sport psychology frameworks that support the needs and preferences of Deaflympic athletes and promote equitable access to effective mental training resources.

## Introduction

The elite sport movement for athletes with hearing impairments, the Deaflympic movement, offers a dedicated platform where this athlete group can compete without the auditory and communicative barriers they face in the hearing world. The competition rules in the Deaflympics closely mirror those of the Olympic Games. Yet, Deaflympic sport include key adaptations such as visual signals replacing auditory cues to meet the communication needs of athletes with hearing impairments (3, 4). The International Committee of Sports for the Deaf (ICSD), the governing body responsible for overseeing Deaf sports globally, regulates that athletes must have a hearing loss of at least 55 decibels in their better ear to compete. The use of hearing aids or cochlear implants (CI) is prohibited during competition to maintain fairness (ICSD, 2009). These regulations allow hard-of-hearing (HH) athletes, who can process some auditory information to a certain extent, to compete alongside athletes with profound hearing loss. In addition, this means that some athletes who rely on hearing aid technologies in their daily lives must adapt to a distinctly different sporting environment in which the use of such devices is not permitted. Unlike the Paralympics, the Deaflympics do not classify athletes based on the degree of their disability, which allows all eligible Deaflympic athletes to compete together under the same conditions. This criterion results in a heterogeneous group of athletes competing in the Deaflympics (5, 6).

The heterogeneity among Deaflympic athletes is profound, shaped by a wide spectrum of hearing loss characteristics, communication preferences, and cultural identities (7, 8). First, they have varying degrees of hearing loss, ranging from complete deafness to severe and moderately severe levels, with some retaining residual hearing. Further, Deaflympic athletes experience different types, etiologies, and onsets of hearing impairment. These varying hearing impairments may influence Deaflympics athletes' communication preferences, psychological development and sport performance in an interplay with additional sociodemographic factors (9). For example, the age at which hearing loss occurs and the type of communication exposure during childhood can have a significant impact on cognitive development, particularly in areas such as language acquisition (10). Deaflympic athletes' diversity extends to their cultural identification. Deaf individuals (referred to with a capital “D”) tend to use sign language as their primary mode of communication and view Deafness through a cultural and communicational lens. On the contrary, deaf individuals (referred to with a lowercase “d”) may align more with the hearing world, relying on spoken language and lipreading. Many of the latter group use hearing aids technologies in their everyday lives (11, 12). Together, these factors create a complex and diverse population of athletes with unique strengths and challenges.

D/deaf individuals are often referred to as “people of the eye”, a term that emphasizes the centrality of visual perception in their lives in contrast to hearing and deafblind individuals. This visual orientation is fundamental to how D/deaf individuals process information, engage in social interactions, and perceive the world around them (10, 13). Relying on their visual sense for communication, Deaf people primarily use sign language, a complex visual-spatial language. This visual reliance extends beyond linguistic communication to encompass broader social and cultural practices or “Deaf culture”, which places significant importance on visual experiences and social cues (14, 15). Consequently, the concept “people of the eye” not only underscores the adaptive strategies that Deaf individuals develop to navigate their environments but also reinforces the distinct cultural identity centered on visual communication (12, 14, 16). Moreover, visual-spatial orientation influences various cognitive functions. For instance, research suggests that Deaf individuals exhibit some enhanced visual-spatial abilities compared to their hearing peers (17, 18). In the realm of sports, competitive Deaf athletes have demonstrated shorter reaction times to visual stimuli than hearing athletes (5). These and other capabilities associated with visual-spatial orientation are often referred to as “Deaf gains” (17). These gains are celebrated in Deaf culture, which rejects the clinical view of Deafness as a physical disability.

Despite the distinct sociocultural characteristics and communication needs of Deaflympic athletes, research in sport psychology concerning this population has remained limited (19). Further, there are no reliable quantitative sport psychological diagnostic instruments available for this population (20). Consequently, little is known about the application of sport psychology practices and lived experiences of DHH athletes in Deaflympic sports (21). The persistent research gaps in sport psychology within Deaf sports hinder the development of evidence-based interventions adapted to the diverse communication needs of athletes with hearing impairments. In response to these research gaps, this study aims to explore Deaflympic athletes' and coaches' perspective on sport psychology practices. Gaining insights into their views and experiences would enable better understanding of the necessary framework for sport psychology consultation adapted to their needs and preferences. Using a qualitative research methodology, this study seeks to effectively and comprehensively gather data on a variety of topics relevant to understanding the role that sport psychology plays in Deaflympic sport. This study was designed to examine how Deaflympic athletes perceive the role of mental training in shaping their athletic careers and to explore their application of psychological skills and techniques. Further, the study examines athletes' and coaches' views on the potential impacts of D/deafness on athletic performance. Furthermore, this research aims to identify factors, athletes and coaches believe could contribute to the success of mental training in Deaf sports. Additionally, the study explores the aspects that Deaflympic athletes consider crucial for effective collaboration and communication among themselves and with hearing professionals.

## Method

### Research paradigm and study design

A qualitative research approach was adopted due to the exploratory nature of the study and its goal of providing a comprehensive representation of the lived experiences and perspectives of an underrepresented group of D/deaf and hard-of-hearing (DHH) athletes on mental training. Qualitative research suits Deaf Studies as it captures lived experiences and cultural nuances while allowing the creation of “Deaf space”, where sign language and visual-oriented interactions can foster authentic, community-centered research (22). Focus group interviews were used for data collection, as this methodology facilitates generating insights into and under-researched phenomena and populations (23). A critical realist philosophical framework was adopted in the construction of the study design, the formulation of the research questions and the data analysis (24). Critical realism aims to generate explanations about the investigated research topic and its underlying mechanism (25). It transcends paradigmatic debates that limit research impact by bridging realist and constructivist–interpretivist approaches, acknowledging the interaction between enduring “real” social structures and the processes by which individuals experience and understand the world (26, 27). Thus, it optimally facilitates the exploration of DHH athletes' unexplored subjective experiences and perspectives on mental training in Deaflympic sports, even as they are interpreted within the constraints and influences of social structures.

### Situating the authors

Our research team comprises three authors, including one woman and two men, all of whom have substantial expertise in consulting with athletes and teams in competitive sports. The first author (AM-G) specializes in the psychological aspects of Deaf sports, combining his research with practical work and possessing a basic proficiency in German Sign Language (DGS). The second author (AM-E) focuses on the psychological dimensions of performance in Olympic sports, with additional expertise in cultural sport psychology. The third author (RS) is a former Paralympic athlete with extensive research experience in disability and Deaf sports spanning several decades. It is important to note that none of the members of the research team are DHH. To address this, we engaged in extensive consultations with DHH Deaf sports stakeholders and researchers prior to conducting this study. In addition, this study is part of a broader research project investigating sport psychology practices within the population of elite athletes with hearing impairments, in continuous collaboration with the German Deaf Sport Association (DGSV).

### Participants

Athletes from the German Deaf Sport Association (DGSV), who meet the participation criteria for the Deaflympics, and their coaches, were eligible for this study. The DGSV manages 16 elite sports departments, including around 160 Deaflympic athletes. Six of these departments were selected for the study. Both individual (e.g., tennis) and team sports (e.g., handball) were included to ensure representativeness across sport types. Additionally, the selected departments were chosen to reflect the diversity in athletes' communication methods, as well as the degrees and types of hearing impairment. The final sample included 23 active elite DHH athletes (*M*_age_ = 33.09; *SD* = 9.47), representing approximately 14.3% of the total Deaflympic athlete population within the DGSV. Four DGSV coaches (25% female) also participated in the study. All athletes exhibited a hearing loss of at least 55 dB (*M* = 91.40 dB, *SD* = 15.76) in the better ear (see Table 1). The majority of them were born with a hearing impairment to hearing parents and had congenital hearing loss. All of them train and compete in mainstream hearing sports in addition to their training and participation in competitive Deaf sports. Nearly all athletes (91.3%, *n* = 21) had experience competing internationally, and the majority (82.6%, *n* = 19) had won medals in Deaf sports at events such as the Deaf European and World Championships or the Deaflympics. The coaches' sample (*M*_age_ = 51.75, *SD* = 1.26) included one Deaf participant who was a native sign language user and one HH participant. The other two coaches were hearing. The average coaching experience was 15.5 years (*SD* = 9.71) in Deaf sports and 18.5 years (*SD* = 12.40) in mainstream hearing sports.

**Table 1:** Sociodemographic characteristics of participants (athletes).

Sample characteristic			
Variable	Category	*n*	*%*
Gender			
	Female	6	26.1
	Male	17	73.9
Hearing loss severity^a^			
	Severe (65 to < 85 dB)	6	27.3
	Profound (85 to < 95 dB)	5	22.7
	Complete (95 dB or greater)	11	50
Hearing loss onset			
	Congenital	16	72.7
	Postnatal	6	27.3
Parents with hearing loss			
	Yes	6	73.9
	No	17	26.1
Use hearing aid technologies			
	Yes	8	34.8
	No	15	65.2
Sports context			
	Team sports	6	26.1
	Individual sports	17	73.9
Worked with sport psychologist			
	Yes	3	13
	No	20	87

*N* = 23.

^a^ In the better ear.

### Procedure

The study was reviewed and approved by Leipzig University's ethics advisory. The study was conducted in accordance with the local legislation and institutional requirements. The participants provided their written informed consent to participate in this study. Recruitment took place in February and March 2021 in collaboration with the DGSV. The DGSV provided explanations about the study and the purpose of the focus group interviews to all its national sport divisions, inviting interested participants to come forward. After receiving a list of interested divisions, the research team initially selected five divisions for the study. However, as data saturation was not achieved for all interview guide topics, a sixth focus group interview was subsequently planned and conducted. Consistent with Braun and Clarke (28) we did not treat saturation as a strict threshold of informational redundancy, but instead evaluated the need for additional data based on whether key interview topics had been sufficiently explored and whether new, relevant patterns were still emerging. The selection process aimed to include DHH athletes from both individual and team sports, reflecting the diversity of the Deaflympic athlete population. Each interview included four to six athletes, and in four of the interviews, the national team coaches participated alongside the athletes. One interview was conducted in person during a national division training camp, while the others were conducted via the University Leipzig's video conference platform videoconference platform BigBlueButton (29). Two sign language interpreters provided simultaneous interpretation for all interviews. The interviews were carried out by the first and third authors between June and August 2021. Before each interview, athletes and coaches were provided with comprehensive information about the study's aims, procedures, and data protection policies. The interviews lasted between 67 and 93 min (*M* = 84.26, *SD* = 9.05). Following the interviews, participants completed a brief, self-constructed questionnaire that gathered sociodemographic data, information on sports participation, and details regarding their hearing status.

### Interview guide

A semi-structured focus group interview guide was developed following Hennik and colleagues (30). The questions were designed to encourage discussion and to gather in-depth comprehensive information on athletes' and coaches' familiarity with applied sport psychology, (e.g., What do you understand by the term “sport psychology”?), experiences with the implementation of psychological skills and techniques (e.g., sometimes athletes feel tension during competition. How do you deal with this tension?), and communication preferences with teammates, coaches and sport psychology practitioners (e.g., How should Deaflympic athletes and hearing sports psychologists communicate with each other?). In addition, the participants were asked to share their interpretations of a finding from previous research within the overall research project (18), which suggests that Deaflympic athletes may use relaxation techniques less frequently than other commonly employed strategies. Participants were encouraged to discuss and share their diverse opinions and experiences in response to each question. Follow-up questions and probes were used to obtain more detailed insights.

The guide was iteratively adapted after each focus group interview as new topics emerged that required further exploration. The adapted guide included questions about the transition of athletes using hearing aid devices from hearing in everyday life and sports to participating in sport without hearing aids during official Deaf sport competitions. An example of such a question is, “A CI user mentioned that without the CI, she is ‘focused differently'. How does one’s focus change without a CI during competitions?”. Other topics included the potential psychological influence of D/deafness on performance (e.g., “Some athletes have reported that their sense of touch becomes very sensitive when they close their eyes. What role does this play in bowling?”) and the implications of visual orientation and communication on psychological skills (e.g., “Some players talk to themselves during a game or competition to motivate or calm themselves. How does this work when someone uses sign language?”).

### Data analysis

The focus group interview data was first anonymized and transcribed verbatim. Following the critical realism paradigm, a six-phase reflexive thematic analysis (TA) was conducted according to the guidelines of Braun and Clarke (2). MAXQDA 2022 (31) was used for data analysis. In the initial phase, the dataset was read and re-read to ensure familiarity and thorough understanding. During this process, the first and third authors made digital notes in MAXQDA, reflecting on significant phenomena and gathering insights for subsequent analysis phases. The dataset was then inductively coded, with text segments labeled to explore participants' perspectives and subjective experiences. Following Braun and Clarke (2), coding was understood as an active and interpretative process. It was led by the research questions and shaped by reflexive insight, with codes developed iteratively through close engagement with the data. In the third phase, the first author tentatively organized the coded segments into broader themes and subthemes related to the research questions. This thematic map underwent several rounds of review and refinement to optimally represent the athletes' perspectives on the relevant issues. The third author acted as a “critical friend” in this process, providing an external perspective on the allocation of raw data into codes and themes (32). The themes represented different dimensions related to the research questions, encompassing each diverse opinions, ideas, and experiences on a broader topic. These themes guided the analysis of participants perceptions of mental training in Deaflympic sports.

## Results

**Figure 1:**
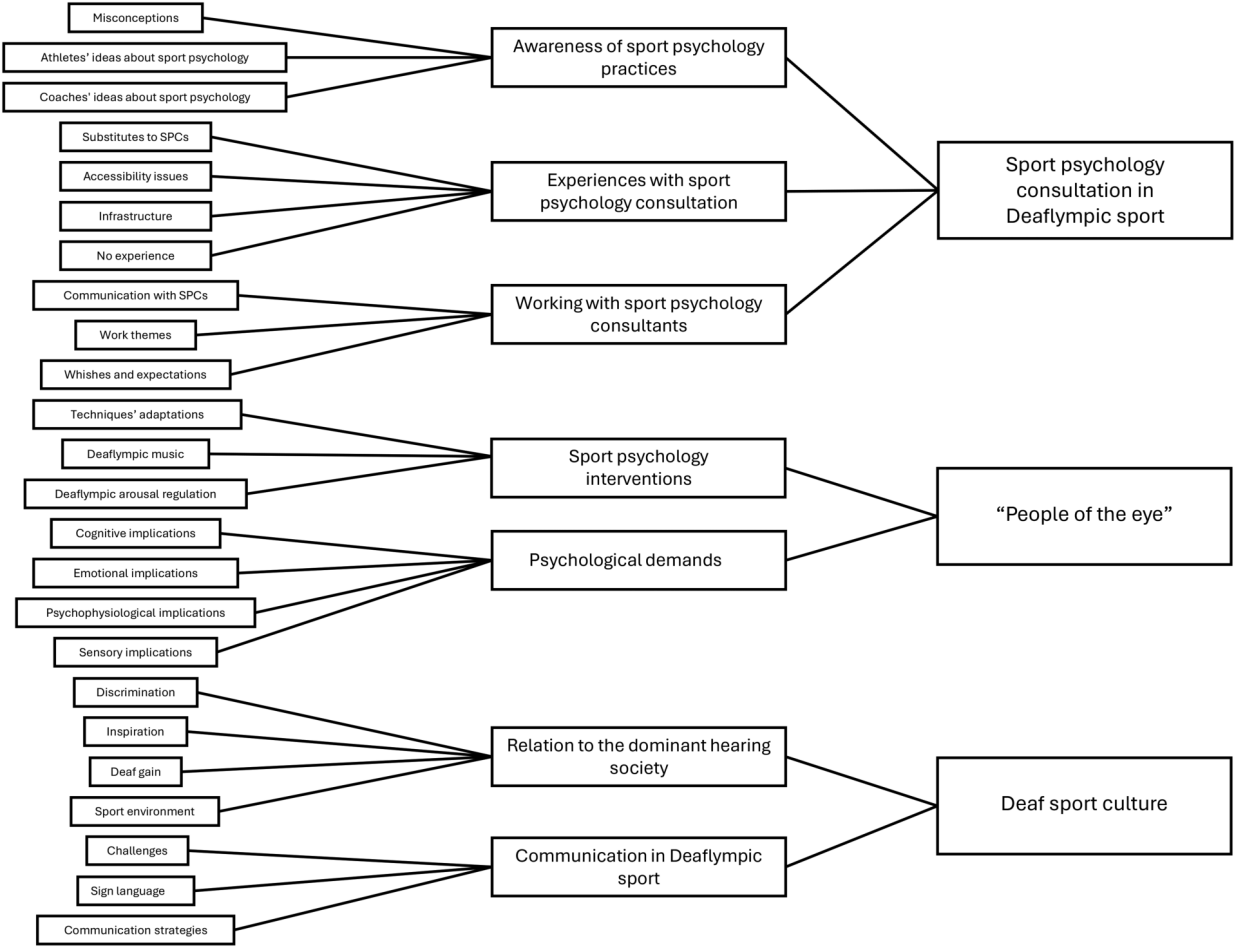
Thematic analysis of Deaflympic athletes' and coaches' perspectives on mental training. Note. On the left are the initial codes, which were grouped into superordinate categories shown in the middle. These categories were further synthesized into overarching themes displayed on the right. Connecting lines indicate the analytic relationships from specific codes to broader categories and themes.

The process of assigning codes to superordinate categories and overarching themes was guided by participants' responses. Initially, 899 codes were generated, reflecting participants' knowledge, perspectives, and experiences regarding applied sport psychology practices in Deaflympic sports. These codes were then grouped into 7 superordinate categories, which were further organized into three overarching themes (see Figure 1). The first theme, “Sport psychology consultation in Deaf sports”, captures participants' perspectives on the applicability of sport psychology consultation in Deaflympic sports. The second theme, “People of the Eye”, represents participants' views on how D/deafness and its associated visual orientation influence sports performance and mental training. The third theme, “Deaf sports culture”, encompasses participants' reflections on the unique characteristics of Deaf sports culture, its relationship with the dominant hearing society, and communication practices in Deaflympic sports.

### Sport psychology consultation in Deaflympic sport

A total of 251 codes were identified regarding participants' perspectives on sport psychology consultation in Deaflympic sports. These were classified into three superordinate categories: Awareness of sport psychology practices, Experiences with sport psychology consultation, and Working with SPCs.

#### Awareness of sport psychology practices

Most participants demonstrated awareness of sport psychology, recognizing various psychological interventions and their potential benefits. One participant explained when asked what they associate with the field of sport psychology: “Sports psychology is a very large field. It's not just about one thing; it involves performance, dealing with pressure, understanding yourself, how to handle frustration, how to manage it, or if you get injured, how to cope with that”. Many participants expressed a strong interest in working with SPCs, outlining clear expectations regarding the aspects they wished to develop, encompassing various areas of applied sport psychology. However, some participants perceived sport psychology as primarily catering to hearing athletes. One participant remarked: “My experience is simply that, for hearing people, if you learn a lot from psychologists at the age of 16, 17, or 18, it is certainly a very valuable and important asset”. Additionally, some athletes indicated a lack of awareness among their peers and coaches about the benefits of sport psychology services in Deaflympic sports. In addition, misconceptions about sport psychology could be detected in some cases. For example, one participant believed that performance improvements require starting psychological training at a young age.

#### Experiences with sport psychology consultation

Despite widespread recognition of the benefits of sport psychology and interest in engaging in consultation, only one participant reported having ever received sport psychology support, and even this was in the context of hearing sports. When participants were asked about the reasons for the lack of SPC engagement in Deaflympic sports, they identified accessibility as a key barrier. First, athletes reported insufficient support provided from national organizations and local clubs, as illustrated by one participant: “There are also very few opportunities for sports psychologists, which is why I don't have one. I reflect a lot on my own, I spend a lot of time in self-reflection, and I basically train myself mentally”. In addition, coaches confirmed the challenges their athletes faced in accessing sport psychology services and described similar difficulties for themselves: “I want to develop as a coach so that I can delegate areas of responsibility to specialists. I am also looking for someone in the field (mental training) who can support me in this area or help improve things”. Furthermore, a significant issue raised by Deaf participants was the lack of sport psychologists who are native sign language users. One participant emphasized the language barrier: “I know a few hearing athletes who go to a sports psychologist, but of course, for me, that would be an important factor—the person would need to know sign language”.

Due to the absence of engagement with SPCs, Deaflympic athletes often relied on alternative forms of support, such as self-reflection or seeking advice from family and friends. One participant described how their partner acted as an informal SPC:

Family can somewhat take on the role of a sports psychologist. For example, my boyfriend was also at a boarding school for a long time and acted as my sports psychologist in many ways…That was really important to me. Now that he's no longer at the sports boarding school, I notice how much that affects me. So, I've realized that family can almost take on the role of a sports psychologist.

Coaches also attempted to fill the gap left by the lack of professional sport psychology services, though their efforts were limited by their lack of training and resources. One coach expressed: “That's why I said I wouldn't be opposed to professional support. I try to use my amateur knowledge to do some sport-psychological things to give the guys a bit of a push…I feel like that works well”. Another coach expressed frustration over financial constraints that prevent personalized support for his athletes.

#### Working with sport psychology consultants

Participants identified various themes, skills, and techniques they wished to work on with SPCs, either as individual athletes working one-on-one with an SPC or as teams engaging in group consultations. Most of these areas were not specific to Deaf sports but reflected general characteristics of competitive sports. One athlete shared: “I often feel like I'm stuck at a certain point in my training and doubt myself too much, thinking that I can't do it. That's why I've now decided to find a sports psychologist—to learn how to be less self-critical”. However, some consultation goals were unique to the context of Deaf sports. For example, one HH team sports athlete, who is a native spoken language user, expressed the need for SPCs to assist with team communication:

When I communicate with a Deaf person, I often feel that I am misunderstood. What I want to say doesn't always come across the way I intend it… If someone were to do this for the entire team, then maybe there would also be tips on how to improve communication among each other…I would expect the psychologist to help with this aspect of communication.

Another topic raised by participants was thse challenge of transitioning between daily life and participation in competitive Deaf sports, particularly given the financial constraints that are prevalent in Deaf sports. Additionally, some Deaf athletes expressed the wish for hearing SPCs to act as a bridge between them and the hearing world: “I believe that deaf people always face many barriers in communication with hearing people, and the sports psychologist should perhaps also take that into account in some way”.

Participants also discussed essential factors for establishing an effective working relationship with SPCs, with communication emerging as a central concern. For some Deaf athletes, the most crucial factor was that the SPC could communicate in sign language: “A hearing person—how is communication supposed to work? Should we write everything down on paper and keep passing it back and forth? No, it has to be sign language”. For some, the SPC's hearing status was irrelevant as long as they could communicate fluently in sign language. Others, however, maintained that only a Deaf SPC could possess the cultural and experiential insight necessary to fully grasp their lived experiences: “If a hearing sports psychologist is working in the Deaf community, they don't really know the world of deaf people. It would have to be a deaf sports psychologist who can understand and empathize with the world of Deaf people”. While some Deaf athletes were sceptical about using sign language interpreters for communication (e.g., “Then it becomes a three-person setup. That can work, but I imagine it to be difficult”), others viewed it more positively and reported good experiences with interpreters. In contrast to native signers, HH athletes expressed a preference for working with SPCs who were either hearing or HH individuals proficient in spoken language, as most of them do not use sign language.

As there were neither D/deaf SPCs nor SPCs who were native sign language users in Germany at the time of data collection, participants were asked how hearing SPCs could establish effective working relationships with DHH athletes and Deaf teams beyond the use of sign language interpretation. First, participants suggested that some SPCs could acquire sign language skills through long-term collaboration with the DGSV, as some association coaches had done. Additionally, the importance of SPCs gaining a deeper understanding of the lived experiences of DHH individuals was emphasized. One participant noted: “They really need to immerse themselves in the life of deaf people. The psychologist needs to understand the life of deaf individuals, and then working with interpreters would be manageable”. Furthermore, participants identified several key factors crucial for establishing trust and rapport with DHH athletes. These included the SPCs' interpersonal skills, respect for and enthusiasm toward Deaf sport culture, communication at eye level, and a genuine commitment to understanding the experiences of Deaf athletes. One participant explained:

It really comes down to trust. I think any deaf person can communicate with a hearing person, whether visually or through spoken language—that doesn't really matter. What's important is that they're on the same page, that the hearing person can really put themselves in the deaf person's shoes and understand them. For example, I remember a coach back then, he just really got deaf people…If we had psychologists like that—especially hearing ones—then it wouldn't be a problem at all. But if you have a psychologist who thinks they’re above you, that would break trust. It’s really all about trust. In that case, it would probably be better to have a deaf or hard-of-hearing psychologist, or at least someone who's completely fluent in sign language. So, in the end, it's not just about whether they're a coach or a psychologist—it's about the kind of person they are.

### People of the eye

392 codes were generated related to athletes' reflections on the influence of visual orientation and/or D/deafness on mental training and sports performance. Participants' reflections were categorized into two superordinate themes: Sport psychology interventions and Psychological demands.

#### Sport psychology interventions

The athletes described the use of various psychological skills and techniques, similar to those employed by Paralympic and Olympic athletes. For example, one athlete described their use of imagery training: “I try to internalize these technical processes…I refine these movement sequences and improve them visually…Refine the technique again, and go through it in my head once more”. Yet, their descriptions highlighted specific adaptations and nuances that emerged in relation to D/deafness in the application of these techniques.

##### Self-talk

Sign language is a visual language, making it challenging for native signers to use it discreetly during competitions. One athlete explained: “It is uncomfortable because others can look too, so I think I wouldn't do that either because I feel like everyone sees it and is looking at me”. To adapt to the visual modality of sign language, Deaf athletes engage in inner dialogues similar to those of hearing athletes. Some described these inner dialogues as visualizing themselves signing, while others characterized them as “images’ or “thoughts”. One athlete noted: “I don’t think in signs. I think, I believe, I think in images, in pictures. For example, by recalling pictures of how I could be relaxed”. Another way native signers conduct self-talk in sign language was by finding a private place to sign without being noticed:

When I'm really under pressure, I very often go to the bathroom, for example—so, into a room where no one can see me. Then I do sign to myself in the mirror and say: That was really bad just now. And I sign it to myself there. Then I feel like I've let it out, and I have a positive feeling afterward. That helps me—I've always done that.

##### Arousal regulation

Many arousal regulation techniques are dependent on auditory cues, such as music. Athletes who use hearing aids reported utilizing music for relaxation and activation purposes, similar to Paralympic and Olympic athletes:

Before a competition or a game, I always listen to music. Yeah, sure, I'm deaf, that's true, but I turn it up really loud. If I didn't have it, I’d play poorly—I already know that. And the music helps me…I use music that's very bass-heavy. I need the bass when I warm up. Before, I always used headphones or my hearing aids to play the music through, and that also boosts my motivation.

In contrast, Deaflympic athletes not using hearing aid technologies reported that the motivating and relaxing effects of music are not available to them. One participant explained: “Well, with hearing people, I often see that they relax by listening to music. But of course, that's not really an option for me”. Instead, athletes reported utilizing various alternative relaxation methods. Some described using visual stimuli and positive memories, such as focusing on familiar objects or recalling comforting images, to facilitate relaxation. Others mentioned engaging in tactile stimulation, such as holding certain objects in their hands applying pressure. Some also highlighted the use of taste-based stimuli, such as sucking on a piece of chocolate, as a means of relaxation. Some athletes highlighted movement as a strategy (e.g., “I also need movement, so I take another lap around the competition venue, try to go outside for a few minutes, and then really let go of these thoughts mentally”). Additionally, several participants emphasized the role of interpersonal communication, noting that engaging with teammates and coaches during competition contributed to their ability to regulate arousal levels.

As noted in the method section, relaxation techniques were found to be used less frequently than other psychological strategies among Deaf athletes (18). Reflecting on this finding, one athlete reported receiving various inputs from different coaches, but none proved effective. Other athletes shared the observation that relaxation techniques were not systematically integrated into their training: “Maybe that's not being trained. the coach doesn't teach the athletes how to control their breathing, focus on themselves, and relax. I could imagine that there is no awareness of this among the coaches”. One coach echoed these concerns, emphasizing the challenges of implementing relaxation techniques in a group with diverse communication needs.

##### Imagery

Similar to relaxation techniques, many imagery techniques are traditionally guided with closed eyes and rely on auditory cues, making them less accessible to Deaf signers. One coach described how they adapted an imagery technique to be inclusive for all players:

When we arrive in a foreign country, we form a circle already at the airport with the idea of imagining that we will form the same circle again when we fly back—with the trophy in hand. Deaf players are even more receptive to this. Then, I ask the players to briefly close their eyes for a few seconds, about ten seconds. I give them a time frame and tell them in advance what they should visualize. Then they close their eyes, and at some point, I give a signal—this can be a clap, knocking on the floor—they feel it immediately, and then it's over.

#### Psychological demands

The participants expressed diverse perspectives on how D/deafness and reliance on visual processing shape psychological demands during training and competition. These differences largely stemmed from variations in auditory processing abilities among athletes, leading to distinct lived experiences within the Deaflympic environment. In particular, two subgroups of Deaflympic athletes emerged. The first group includes athletes who do not use hearing aids and are therefore accustomed to navigating the world without auditory input. The second group consists of athletes who rely on auditory information in their daily lives with the assistance of hearing aids. For them, the Deaflympic environment represents a significant shift, as the prohibition of hearing aids in competition forces them to adapt to a fully non-auditory setting, which differs from their usual communication and sensory experiences.

##### Stress and nervousness

Some athletes who rely on sign language reported experiencing heightened stress due to their visual orientation:

I'm not relaxed at all, I'm actually always on high alert. Because I take in everything with my eyes. My energy is constantly flowing. Hearing people have the auditory sense and can close their eyes to relax—that doesn't work for me. So, I'm basically always in a state of alertness. There's really nothing truly relaxing for me. I would say I constantly need energy.

A coach reflects on the increase in stress levels among HH and deaf athletes while competing in the Deaflympics without the use of their hearing aids and the resulting changes in their communication methods: “The sports medical department always points out that our players have incredibly high muscle tension compared to other to hearing athletes. I find that interesting, but I can't quite explain where that comes from”. Another coach reflected on this phenomenon and described changes in their communication means:

You can see a certain helplessness in the players at first. They have to assess the situation and figure out what’s happening. As soon as the hearing aid is turned off, especially with CI users, you notice that they become more restless. They seem more stressed at first. In general, the players have to make a much greater effort. They need to use their peripheral vision more extensively. They have to find the right balance between looking at the bench, their teammates, and the overall game. Overall, everything becomes more stressful, and that's why they feel the need to touch someone—to physically feel another person in order to regain a sense of presence in their surroundings.

However, other participants did not perceive any direct impact of visual orientation on stress levels, instead attributing stress to individual personality traits rather than D/deafness.

##### Sense compensation

Some participants described how, in addition to their reliance on visual perception, their other senses become more attuned during sports activities and competitions:

We are visual people. Sure, we don’t have the sense of hearing, but we perceive a lot more visually…When I close my eyes, I become extremely tactile-sensitive, much more aware of sensations. Our lack of hearing is simply compensated for by our other senses. I suddenly become much more aware of how something tickles my feet, and when I close my eyes, I notice: “Oh no, now it's tingling here and there".

This heightened sensitivity was viewed by some as an advantage, particularly in relation to bodily awareness and responsiveness. Others, however, described it as a challenge, as external stimuli could become overwhelming and distracting. One participant explained:

When I take the (bowling)ball in my hand and concentrate, and then, on the lane next to me, for example, someone suddenly drops a ball—it's not just a visual distraction for us. There's also the vibration, the disturbance on the ground. I was just focused, and suddenly, a ball drops next to me on the lane. Everything that enters our field of vision can be distracting. A camera, for example, or if someone waves a flag, or anything else—it can all be distracting.

##### Concentration and attention

As illustrated in the previous quote, participants noted that their visual orientation could impact their ability to concentrate, sometimes impairing performance. One athlete elaborated: “We, as deaf individuals, we are so visually oriented that we immediately notice when something moves—a light turning on or off, or people moving. That can really be distracting and can also affect my performance”. A potential explanation for this phenomenon, as provided by participants, is the increased attentional demands associated with relying exclusively on visual cues. Unlike hearing athletes, who can orient themselves using auditory cues, such as the sound of a ball moving across the grass, DHH athletes must constantly scan their surroundings, which requires greater cognitive effort. One participant described this heightened attentional demand:

Actually, you could say that you hear with your eyes. You have to take in a lot of impressions and be even more attentive because verbal cues are rare. Most people don't hear them, so you have to see what's happening. If someone announces something, you absolutely have to notice it visually. That's why you have to be much more alert. For example, if you look to the left and your teammate calls something out, a hearing person would notice it. But for us, we have to constantly look left and right to really catch everything.

However, some participants saw their reliance on visual information as an advantage, particularly when interpreting movement and game dynamics: “Since we are visual people we might perceive the game differently…I would say that sometimes I can read the game better than other players because I can clearly see how the opponents are moving”.

HH and deaf athletes relying on hearing aids in their everyday life reported difficulties in adapting to the transition from auditory referee cues (e.g., whistles) in hearing sports to visual cues (e.g., flags) used in the Deaflympics. This shift requires conscious effort and sometimes leads to delayed reactions in their experience. Further, some athletes described they sense a qualitative shift in their focus when using or not using hearing aids, although they struggled to articulate exactly how it changes. Others reported no difference in the level or quality of concentration.

##### Emotions

HH and deaf athletes relying on hearing aids also shared the experience that the inability to hear during competition affects their sense of presence and connection to the sporting environment:

I am HH, and when I play football with hearing players, I always use my hearing aids. I play with them and can hear everything: the fans, the crowd and everything else. I'm used to it. But when I switch to Deaf football and turn off my hearing aids, I sometimes feel a sense of emptiness. I can still see the spectators and their facial expressions, but I can't hear them anymore. It's a completely different feeling, and I notice that something is missing. At the same time, I still perceive emotions very strongly, because I see facial expressions and how people are approaching me. It's almost like images rushing toward me—it's a very different perception.

### Deaf sport culture

The final theme in the analysis encompassed participants' perspectives on cultural aspects of Deaf sports. A total of 121 codes were generated, which were categorized into two overarching areas: Relationship with the dominant hearing society and Communication in Deaflympic sports.

#### Relation to the dominant hearing society

Participants frequently compared their experiences in hearing sports with those in Deaf sports. Some Deaflympic athletes described facing discrimination in hearing sports, and even, in rare cases, within Deaflympic settings from hearing coaches and professionals. These experiences included perceptions that DHH athletes were not as competent as their hearing counterparts, as well as a general lack of awareness or effort from professionals to accommodate their needs. Jenny (pseudonym), for example, reflected on how her hearing coach and training partners assumed that sport psychology services were only relevant for hearing athletes: “I had a mental coach when I trained with hearing people…I remember that, in the past, whenever I went to mental training, they would always say: ‘Oh no, we can't practice with Jenny because she's deaf’”

However, not all experiences with hearing sports were negative. Some participants, including both hearing aid users and sign language users, reported positive engagement with hearing professionals and teams.

Many emphasized that their participation in Deaflympic sports provides them with an environment where their communication needs, hearing status, and Deaf cultural identity are recognized and respected. One participant described the unique atmosphere within their Deaf national team:

When we're in the locker room, the hearing aids are turned off, and the CIs are taken out, so everything is communicated solely through sign language—lip reading, sign language—and at that point, we are all on the same level, so to speak. That's also the essence of Deaf teams…And I think in our deaf national team, or in general, it is a must to sign or at least to make an effort to communicate with your hands.

Despite this sense of inclusion, some athletes highlighted tensions arising from differences in communication preferences and hearing abilities. For instance, an athlete who uses CI noted:

Thanks to my CI, I don't have any language barriers when communicating with hearing people. For Deaf people who rely on sign language, communicating in the outside world is, of course, much more challenging. That's why the Deaflympics exist—a community where deaf people come together and participate in activities. In those moments, I sometimes actually feel like an outsider.

#### Communication in Deaflympic sport

Participants in team sports reported that the majority of their teammates rely on hearing aid technologies and have limited or no proficiency in sign language. This generational shift, due to advancements in hearing aid technologies, has led to tensions in Deaf teams regarding communication. On the one hand, these teams are part of Deaf sports, which traditionally prioritize sign language as the primary means of communication. On the other hand, with fewer athletes now relying on sign language, team communication strategies must evolve to accommodate a diverse linguistic landscape. A 40-year-old athlete reflected on these changes over time:

When I started, I was one of only two-three people who were HH. The rest were deaf, so I was practically an outsider because everyone communicated only in sign language. I could only talk to one or two others because it was difficult to communicate with the deaf players. But they always tried to include me so that I could learn. Over time, I picked up more sign language. Over time, people started speaking more. I think now there are one or two people in the team who know sign language, but the rest don't. And then we all come together and are supposed to communicate, even though almost no one knows sign language.

Athletes who rely on spoken language in their daily lives reported the necessity of developing alternative communication strategies while competing in Deaf sports alongside Deaf athletes. Given the absence of auditory cues, they have to adapt their communication methods dynamically during both training and competition:

In hearing teams, many people say that I work much more with physical contact. For example, when I'm in the block, I use my hands in the circle so that I know where my teammate is. Since I can’t rely on sounds as well, like when someone is behind me, I prefer to keep a hand in contact with the circle, so I always know where they are. And specifically in Deaf sports, I've realized that I automatically rely much more on my eyes—especially when it comes to communication with other players. Also, with the coach, I have to actively seek eye contact. And when calling out plays, I need to visually confirm that everyone has understood what I announced… As a central player in a Deaf team, I have the extra responsibility to make sure everyone gets the message and gives me a quick visual confirmation.

Deaf teams develop various strategies to foster effective communication and ensure all players feel included. First, many athletes reported adapting their communication style based on their teammates' preferences and abilities “You naturally notice which form of communication works and adapt to it. If it works well with Person A, that doesn't automatically mean it will work the same way with Person B”. Additionally, some teams developed a unified signing system on the pitch, coordinated with the coach, to streamline communication during games: “Sign language alone isn't enough…You need short, clear signals so that everyone immediately understands what's meant. Full sentences don't work in that setting. Just like when you shout something on the field, you shorten it—it's the same with signs”. Another widely used strategy involved memorizing and coordinating plays before games. Some Deaf athletes reported that they spent extra time learning their teammates' playing styles so they could anticipate their movements without needing verbal confirmation. Body posture was also highlighted as a crucial nonverbal communication tool: “Whether you try to communicate with signs or gestures, or simply use body language—often, your overall presence already helps a lot. For example, if you want to say, “Pick up the pace!”, body posture can be really important”. Beyond specific strategies, participants emphasized that fostering familiarity and trust within the team was fundamental to effective communication and overall team cohesion. Establishing strong interpersonal connections allowed players to navigate communication challenges more effectively and create a sense of unity on and off the field.

## Discussion

The findings of this study provide new insights into the perspectives of Deaflympic athletes and coaches on mental training. They reveal key aspects of sport psychology consultation in Deaflympic sports, including the influence of visual-spatial orientation on psychological skills and demands, as well as the role of Deaf sport culture in shaping athletes' experiences.

### Sport psychology consultation in Deaflympic sport

While Deaflympic athletes demonstrate both an awareness of and a strong interest in sport psychology, their engagement with professional support remains highly limited. Both athletes and coaches identify accessibility barriers and the lack of culturally adapted services as the primary obstacles to utilizing sport psychology services in Deaflympic sports. This aligns with the current state of sport psychology in Germany, where no DHH SPCs are native sign language users (1). However, the absence of SPCs fluent in sign language does not fully explain the lack of psychological support available to HH and deaf athletes who rely on spoken language with the assistance of hearing aids. These athletes expressed a preference for working with SPCs who are either hearing or fluent in spoken language. For them, financial constraints and the limited federal support for Deaflympic sports likely present more significant barriers (3).

For Deaf athletes, however, communication in sign language is a crucial factor in addition to financial constraints when accessing effective psychological support. These athletes prefer working with SPCs who are proficient in sign language and familiar with the cultural and perceptual realities of Deaf individuals. This preference aligns with research emphasizing that effective psychological support relies on culturally competent professionals who understand the lived experiences of their clients (33, 34). The importance of cultural competence in sport psychology is particularly recognized in work with culturally marginalized communities and athletes with physical disabilities (35, 36). For Deaf athletes, trust is built not only through sign language communication but also through an SPC's willingness and ability to engage with Deaf culture and understand the broader sociocultural realities they navigate (37).

To bridge the linguistic gap between Deaf athletes and hearing professionals, sign language interpretation is a viable option for some but not for others. These findings align with research on the preferred communication methods of DHH individuals in clinical settings, which found that approximately half of native sign language users prefer to communicate through sign language interpreters. Others, however, prefer direct communication with professionals who can sign, while a smaller group is willing to communicate with professionals using spoken language, provided they are familiar with Deaf sociocultural issues (38). The scepticism some Deaf participants expressed toward interpretation is consistent with existing literature, which suggests that sign language interpretation does not always fully meet the needs of Deaf clients. Interpretation involves a triadic setting—where communication occurs between the client, the interpreter, and the consultant—which can lead to misunderstandings, misdiagnoses, and even mistreatment, particularly in medical and psychological services (39, 40). This concern is even more pronounced among a subgroup of Deaflympic athletes who cannot envision working with an SPC unless the consultant is D/deaf themselves.

Apart from accessibility challenges, some Deaflympic athletes view sport psychology as primarily designed for hearing athletes, and misconceptions about its relevance persist. Such attitudes may contribute to the continued stagnation of sport psychology services in Deaf sports (1, 41). Deaflympic athletes therefore develop alternative strategies to enhance their mental resilience. They seek support from friends and family, draw inspiration from hearing peers in competitive sports, and rely on their coaches' mental training expertise. When given the opportunity to work with SPCs, Deaflympic athletes have clear expectations regarding the support they require. These expectations go beyond general performance optimization and well-being, as they also reflect specific needs unique to DHH athletes. This study identified two key areas where athletes seek specialized support: facilitating communication and cultural understanding between the Deaf and hearing worlds and optimizing communication within Deaf sports teams. Currently, these issues are neither addressed in sport psychology handbooks [e.g., (42)] nor explored in sport psychology research, and they are also notably absent from the education of practitioners (1). As a result, SPCs working in Deaflympic sports presumably lack the knowledge about interventions needed to effectively address these challenges.

### People of the eye: psychological skills and demands

The unique visual-spatial orientation of Deaflympic athletes influences various psychological and communication factors in competitive sports, as well as the way certain sport psychology interventions are applied. While participants' descriptions suggested that their use of these techniques was largely identical or similar to those observed in hearing sports, specific adaptations and considerations unique to Deaflympic sport were identified. This finding aligns with existing sport psychology research in Paralympic sports, which emphasizes that interventions can and should be tailored to meet the unique needs of athletes with physical disabilities (43, 44). Similarly, DHH athletes adapt psychological skills and techniques to align with their linguistic and sensory experiences. For example, they may employ internal visualizations or engage in private signing in secluded spaces as self-talk techniques. Additionally, they described different language modalities they engage with in their inner dialogues, including sign language, spoken language, and more abstract constructs such as “inner images” and “thoughts”. Research supports the idea that inner dialogue in D/deaf individuals is multimodal, incorporating visual, proprioceptive, and motor components rather than being based on auditory speech (45). However, it remains unknown how this inner-dialogue modality relates to the effectiveness of self-talk in Deaflympic athletes.

The use of music as an arousal regulation method is common in Deaflympic sports, a finding that may seem counterintuitive to hearing individuals. However, Deaflympic athletes, including those with profound hearing loss, can access music through hearing aid technologies. In contrast, D/deaf athletes who do not use hearing aids rarely reported using music. Research suggests, however, that D/deaf individuals can engage in “multisensory listening,” experiencing music through tactile, visual, and kinaesthetic means (46, 47). This raises the question of whether multisensory listening could be utilized as a sport psychology intervention for Deaflympic athletes without hearing aids. For example, Vibrational Music Therapy (VMT) offers a potential alternative, allowing athletes to perceive music through bodily vibrations. VMT also integrates non-verbal communication techniques, such as social-haptic communication, to enhance accessibility for D/deaf and DeafBlind individuals (48). Notably, haptic communication is already used in Deaflympic sports, both as a communication method among teammates and as a coaching tool for imagery interventions. This suggests its potential for broader sport psychology applications in Deaflympic sports.

In addition to or instead of using music, Deaflympic athletes report employing alternative relaxation techniques that engage their other senses. However, there is currently no research on the effectiveness of these intuitive methods or on adapted sport psychology relaxation techniques for Deaflympic athletes (Anonymized). Addressing this gap is crucial, as DHH individuals face additional stressors in their daily lives compared to their hearing peers, including communication barriers, discrimination in education and workplaces, and limited access to healthcare and mental health services (49, 50). These heightened stress levels are not only evident in daily life but also extend to sport contexts. This is supported by both self-reports and a coach's observations of physiological assessments, which indicated increased muscle tension in DHH athletes compared to their hearing counterparts, suggesting elevated physical stress responses. This study therefore highlights the urgent need for research on relaxation techniques tailored to Deaflympic athletes.

The findings on heightened stress levels suggest that D/deafness may influence various psychological factors in sports beyond its impact on how psychological skills and techniques are used and adapted. Participants' experiences indicate that some challenges, such as stress and distractibility, may be linked to the heavy reliance on visual cues in the Deaflympic sport environment. Without or with limited access to auditory information, Deaflympic athletes must process visual cues to orient themselves, communicate with teammates, track the ball, and receive referee signals, to name a few. This intense visual dependence poses significant challenges, particularly for HH and deaf athletes who rely on hearing aids in daily life.

In addition to visual reliance, participants reported using memorization of tactical moves to bypass communication difficulties. This places additional demands on their working memory, requiring them to manage more cognitive tasks than hearing athletes. As a result, their reported struggles with stress and distractibility during competition may stem from increased cognitive load—the mental effort needed to process, interpret, and retain information (51, 52). Prolonged exposure to high cognitive load in sports may lead to cognitive fatigue, a state that temporarily impairs sustained attention, increases distractibility, and reduces decision-making efficiency (53, 54). Some Deaflympic athletes and coaches described experiences aligning with symptoms of cognitive fatigue. This finding corresponds with research on cognitive fatigue in e-learning, showing that DHH students experience higher cognitive and visual fatigue due to their reliance on visual processing, leading to increased stress and mental exhaustion compared to their hearing peers (55).

### Deaf sport culture and communication

Deaflympic athletes and coaches acknowledge the challenges of visual-spatial orientation in competitive sports but also recognize its advantages. For example, they believe Deaflympic athletes have a better understanding of team tactical drills than their hearing counterparts. This belief aligns with research suggesting that D/deaf individuals possess enhanced visual-spatial abilities (56). It also reflects the concept of Deaf gain, which highlights the benefits and unique contributions of Deaf culture and visual-spatial orientation to cultural diversity and enrichment (17). At the same time, DHH athletes reported experiences of discrimination, isolation, and stigmatization in hearing sports due to their hearing status, a phenomenon known as Audism (57). These challenges have also been documented in studies examining the experiences of Deaflympic athletes in hearing sports (58). From athletes' experiences, it is evident that Deaflympic sports serve as a safe Deaf cultural space where they can compete without facing such biases.

Although sign language is central to Deaf culture and celebrated in the Deaflympic sport movement, its practical use in Deaf team sports is declining, a trend also supported in the literature (9). The increasing use of hearing aids among German Deaflympic athletes reflects a broader pattern in Western societies, where advancements in hearing aid technology have made them more accessible. More Deaflympic athletes are now educated in inclusive classrooms where spoken language is the primary mode of instruction, rather than in Deaf schools, as was common in the past (10, 59). Given these shifts and the lack of a shared language among all athletes, Deaf teams must find alternative ways to communicate in a mutually understood, coordinated, and effective manner. This study presents novel findings on diverse strategies used in Deaf sports teams, such as agreed signs and body contact. These findings align with research on collegiate DHH athletes in hearing sports, where teams developed secret sign systems for specific drills (58). However, despite these adaptations, participants reported ongoing communication challenges and emphasized the need for professional support from SPCs in this area.

### Practical implications

The findings of this study offer several practical implications. First, SPCs should consider the visual-spatial orientation of Deaflympic athletes when designing intervention strategies. For instance, tactile and visual cues can replace auditory signals in imagery training. Additionally, since Deaf athletes rely heavily on visual cues for orientation during competition, they may be more susceptible to cognitive fatigue—an important factor to address in mental training. However, it is crucial not to pathologize their visual-spatial orientation, as it holds significant cultural value for culturally Deaf individuals. The study highlights the importance of SPCs respecting Deaf cultural values and immersing themselves in the world of DHH athletes to build trust and rapport. Moreover, visual-spatial orientation presents not only challenges but also potential advantages, such as enhanced visual-spatial skills. SPCs working with DHH athletes can leverage these unique capabilities.

Furthermore, the study emphasizes the importance of allowing Deaflympic athletes to independently choose their SPC and, if desired, their interpreter, to ensure a trusting relationship and the ability to communicate in their preferred language (60). Sign language interpretation alone cannot fully bridge communication gaps between hearing SPCs and Deaf athletes in all cases, as it presents various challenges and should therefore not be assumed as a fully satisfactory solution (61, 62). Additionally, when SPCs work in Deaf sports, addressing communication within Deaf teams is essential for providing meaningful support. Moreover, given the limited availability of SPCs who are native signers, organizations such as the DGSV, the German Society of Sport Psychology (asp), and the German Federal Institute for Sport Science (BISp) should collaborate, support, promote, and fund cultural education and sign language training for SPCs seeking to work in Deaflympic sports. To increase SPCs' involvement in Deaf sport, training programs should include Deaf-specific content. In addition, funded placements or fellowships from international or national organizations could provide SPCs with hands-on experience. Finally, joint practice-based research projects can help promote Deaf sport as a valuable and innovative field within sport psychology.

### Limitations and future research directions

Despite this study's significant contribution to the field of sport psychology in Deaf sports, it has several limitations. First, the research team was composed entirely of hearing researchers. Although DHH researchers and Deaf sports stakeholders were consulted during the study's planning, data collection, and analysis, some Deaf cultural perspectives may have been overlooked. To ensure a more inclusive and culturally informed approach, future research in Deaflympic sport psychology should be pursued internationally, with efforts made to collaborate with D/deaf sport psychology researchers as core members of the research team (63). Second, while this study reveals how Deaflympic athletes employ and adapt psychological skills and techniques, it does not assess their effectiveness. Future empirical research should explore the impact of these adaptations on athletic performance and psychological well-being. Third, future studies should focus on developing and evaluating sport psychology interventions tailored to Deaflympic athletes, addressing their visual-spatial orientation and diverse communication needs.

Given reports of increased tension and stress due to heightened cognitive load during competition, this phenomenon and its underlying mechanisms should be empirically examined. Research should investigate sport-specific stressors in Deaf sports, including cognitive fatigue (64). Understanding these factors in the context of Deaflympic sports will help identify effective strategies to enhance athletes' well-being, performance, and enjoyment of sport. A valuable starting point would be the development and assessment of adapted relaxation techniques suited for Deaflympic athletes. Inspiration could be drawn from the literature on VMT (48). Further, relaxation techniques incorporating tactile and visual cues should be explored. For example, progressive muscle relaxation [PMR; (65)] could be adapted for Deaf athletes and tested for effectiveness. Such adaptations would contribute to refining mental training in Deaf sports, ensuring that interventions align with athletes' communication preferences and sensory modalities. To evaluate such interventions, future studies should incorporate physiological measures alongside accessible self-report tools like visual analogue scales to help bypass communication barriers. Standardized psychological instruments may require linguistic and cultural adaptation, including professional sign language translation, to ensure validity. Moreover, given the limited national pool of eligible participants, cross-national collaboration will be essential to support adequately powered research.

## Conclusion

This study provides novel insights into the use, adaptation, and implementation of psychological skills and techniques in Deaflympic sports. It reveals communication strategies employed by Deaflympic athletes, coaches, and teams, identifies sport psychology themes unique to Deaf sports, and offers practical implications for future research and professional practice. The findings serve as a foundation for adapting and evaluating sport psychology interventions tailored to the communication preferences and visual-spatial orientation of Deaflympic athletes. Moreover, the study demonstrates that Deaflympic athletes are actively engaged with sport psychology practices and seek professional support, just as their Paralympic and Olympic counterparts do. It is now essential for the sport psychology research community to equip SPCs with the tools to work effectively on “eye level” with Deaflympic athletes, coaches, and associations. By addressing and adapting sport psychology practices for Deaflympic sports, the field can ensure that Deaf athletes receive the specialized psychological support they need and deserve to thrive in their sporting careers.

## Data Availability

The datasets presented in this article are not readily available because Alhough the data have been anonymized following standard procedures, the population of Deaflympic athletes in Germany is small, which may pose a risk of identification. Therefore, the interview content (study data) will only be shared after careful consideration and consultation with all co-authors. Requests to access the datasets should be directed to alon.markovglazer@oth-regensburg.de.
